# Editorial: Aromatic amino acid metabolism–Volume II

**DOI:** 10.3389/fmolb.2022.1122151

**Published:** 2023-01-10

**Authors:** Jianyong Li, David J. Merkler, Qian Han

**Affiliations:** ^1^ Department of Biochemistry, Virginia Tech, Blacksburg, VA, United States; ^2^ Department of Chemistry, University of South Florida, Tampa, FL, United States; ^3^ Laborator of Tropical Veterinary Medicine and Vector Biology, School of Life Sciences, Hainan University, Haikou, China; ^4^ One Health Institute, Hainan University, Haikou, China

**Keywords:** aromatic amino acids, metabolism, tyrosine, catecholamine, tryptophan, dopamine, kynurenine, serotonin

Aromatic amino acids (AAAs) are integral components for protein production and their essentiality goes without saying. Moreover, their derivatives often are used by animal species as neurotransmitters that play some specific functions. The evolvement of specific physiological functions in AAA derivatives is likely due to the relative stability of their bulky planar ring structures of these AAAs. As a result, AAAs are structurally much less flexible than most of other amino acids (Li et al., 2021). Although functionally important, animal species cannot produce AAAs. Several essential enzymes required for AAA biosynthesis were either lost in animals or the AAA synthetic pathway never completely evolved in animals. Plants and microorganisms can produce the AAAs, but their production is energetically expensive and, therefore, it is advantageous for animals to obtain them from food sources.

Obtaining sufficient amounts of the AAAs from food sources usually is not a major concern in animal species, especially for humans, but regulation of their individual contents and their metabolites is considerably important to human health. Consequently, disorder of their metabolic pathways often leads to medical issues in humans. Among the three commonly considered AAAs (based on structures, histidine also is an AAA, but it is not produced through the shikimate pathway and not considered a typical AAA), phenylalanine contains a benzene ring, tyrosine has hydroxylated benzene ring, and tryptophan consists of an indole ring. In general, the benzene ring is more stable than indole ring; thus, both phenylalanine and tyrosine are more stable than tryptophan. Because animal species can obtain AAAs from food sources, the ability to breakdown the AAAs or to convert these molecules to other compounds is often important to animal species, particularly humans.

Phenylalanine is relatively abundant amino acid in proteins. In animal species, the major use of phenylalanine is for the biosynthesis of proteins; excess of phenylalanine beyond requires that it be degraded. Animals express phenylalanine hydroxylase, an enzyme that catalyzes the hydroxylation of phenylalanine to tyrosine. Phenylalanine hydroxylase has a moderate affinity to this amino acid (K_m_ ∼ .5 mM), which prevents phenylalanine from accumulating beyond physiological level, but also leaves adequate phenylalanine content for protein synthesis. It is well known that deficiency of phenylalanine hydroxylase leads to a life-threatening disease, phenylketonuria (van Spronsen et al., 2021). Moreover, phenylalanine hydroxylase has high substrate specificity. This characteristic (often overlooked) is very important for maintaining physiological conditions of human and perhaps other animal species and will be briefly elaborated when mentioning tyrosine metabolism.

Tyrosine is moderately abundant residue in proteins and tyrosine derivatives have essential functions in animal species, particularly humans. The presence of a hydroxyl group on the ring somewhat weakens the benzene backbone structure and most animal species can completely catabolize this amino acid to carbon dioxide and water and its nitrogen is incorporated into glutamate by transamination with α-ketoglutarate. Tyrosine can be hydroxylated to L-dopa by tyrosine hydroxylase and L-dopa can be further decarboxylated to dopamine. Dopamine is used for the biosynthesis of norepinephrine and epinephrine. Tyrosine hydroxylase is a key enzyme for synthesis of dopamine and other dopamine derivatives. This enzyme shares extremely high sequence similarity to phenylalanine hydroxylase and is capable of hydroxylation of both phenylalanine and tyrosine, but phenylalanine hydroxylase has no detectable activity towards tyrosine. This is physiologically important in preventing the formation of L-dopa in peripheral tissues because L-dopa oxidizes easily to a catechol moiety under physiological conditions, leading to high oxidative stress and formation of other toxic compounds. Tyrosine hydroxylase expression is limited to only a few tissues, one example being the substantial nigra of the brain. Dopamine (a catecholamine) functions as a neurotransmitter in the brain and the levels of both dopamine and L-dopa must be tightly controlled to prevent their toxic effects. In fact, the toxic effect of the catechol moiety is visualized by the gray color of the substantial nigra in older animals. It is difficult to completely prevent L-dopa/dopamine oxidation. As time passes, the substantial nigra progressively becomes grayer due to catecholamine oxidation. Parkinson’s disease likely occurs if most of the L-dopa producing cells die due to catecholamine oxidation. When enough attention is paid to maintain adequate reducing capability, balanced diets, etc., some L-dopa producing cells, if not most, should survive and remain functional for the entire lifespan of given species.

Tryptophan is the least stable AAA because the indole ring is much less stable than phenyl ring. Tryptophan is relatively rare in proteins and, typically, animals can obtain sufficient quantities from their diet. In humans, majority of tryptophan is degraded through kynurenine pathway. Because it is less abundant, yet necessary for protein production, mammalian species often upregulate tryptophan breakdown during infections as a strategy to starve/eliminate microbial pathogens (Yao et al., 2011). Some tryptophan derivates, such as serotonin and melatonin, are critical for the wellbeing of humans (Richard et al., 2009). Under some pathological conditions, alterations in its breakdown pathway (the kynurenine pathway) may occur and cause various disorders (Fathi et al., 2022).

Although some tyrosine derivatives (such as L-dopa and dopamine) and some tryptophan derivatives (such as 5-hydroxytryptophan and serotonin) are more easily oxidized and may increase oxidative stress, the benefits of their derivatives (i.e., dopamine, norepinephrine, epinephrine, serotonin, melatonin, etc.) to human health and wellbeing far overweigh their potential toxicity under non-health related conditions. In fact, AAAs rarely cause major health issues themselves, but, often, the problems are from disorders in their respective pathways for their biosynthesis, degradation, or modification. In this Research Topic of *Aromatic Amino Acid Metabolism, Volume II*, there are original research articles discussing some positive relationships between increased AAAs and elevated oxidative stress, which is based on increased levels of products under common oxidative conditions (Bagheri et al.), a review article concerning *N*-acetylated AAAs in relation to endocannabinoid anandamide production (Bhandari et al.), another review article concerning the role different tryptophan oxidative enzymes play in tryptophan hemostasis (Klaessens et al.), and two other original research articles: one addressing metabolic profile changes in alcohol-dependent inpatients (Zhu et al.) and another concerning diabetic retinopathy patients (Guo et al.). In both disease conditions, noticeable changes in tryptophan metabolites were reported. Results of these studies revealed some intricacy in AAA metabolism on maintaining physiological condition in living species and suggest that we still have much to learn about AAA metabolism. Overall, researchers are continuously making good progress in the area of AAA metabolism and their efforts should eventually lead to a comprehensive understanding AAA metabolism.
